# Identification of quorum sensing-regulated *Vibrio fortis* as potential pathogenic bacteria for coral bleaching and the effects on the microbial shift

**DOI:** 10.3389/fmicb.2023.1116737

**Published:** 2023-02-03

**Authors:** Xiaohui Sun, Yan Li, Qian Yang, Han Zhang, Nuo Xu, Zheng Tang, Shishi Wu, Yusheng Jiang, Hala F. Mohamed, Danyun Ou, Xinqing Zheng

**Affiliations:** ^1^College of Chemical Engineering, Huaqiao University, Xiamen, China; ^2^Third Institute of Oceanography, Ministry of Natural Resources, Xiamen, China; ^3^Botany and Microbiology Department (Girls Branch), Faculty of Science, Al-Azhar University, Cairo, Egypt; ^4^Key Laboratory of Marine Ecological Conservation and Restoration, Ministry of Natural Resources, Xiamen, China; ^5^Observation and Research Station of Coastal Wetland Ecosystem in Beibu Gulf, Ministry of Natural Resources, Xiamen, China

**Keywords:** coral bleaching, *Vibrio fortis*, symbiotic microbes, bacterial infection, microbial shift

## Abstract

Coastal pollution, global warming, ocean acidification, and other reasons lead to the imbalance of the coral reef ecosystem, resulting in the increasingly serious problem of coral degradation. Coral bleaching is often accompanied by structural abnormalities of coral symbiotic microbiota, among which *Vibrio* is highly concerned. In this study, *Vibrio fortis* S10-1 (MCCC 1H00104), isolated from sea cucumber, was used for the bacterial infection on coral *Seriatopora guttatus* and *Pocillopora damicornis*. The infection of S10-1 led to coral bleaching and a significant reduction of photosynthetic function in coral holobiont, and the pathogenicity of *V. fortis* was regulated by quorum sensing. Meanwhile, *Vibrio* infection also caused a shift of coral symbiotic microbial community, with significantly increased abundant Proteobacteria and Actinobacteria and significantly reduced abundant Firmicutes; on genus level, the abundance of *Bacillus* decreased significantly and the abundance of *Rhodococcus*, *Ralstonia*, and *Burkholderia–Caballeronia–Paraburkholderia* increased significantly; S10-1 infection also significantly impacted the water quality in the micro-ecosystem. In contrast, S10-1 infection showed less effect on the microbial community of the live stone, which reflected that the microbes in the epiphytic environment of the live stone might have a stronger ability of self-regulation; the algal symbionts mainly consisted of *Cladocopium* sp. and showed no significant effect by the *Vibrio* infection. This study verified that *V. fortis* is the primary pathogenic bacterium causing coral bleaching, revealed changes in the microbial community caused by its infection, provided strong evidence for the “bacterial bleaching” hypothesis, and provided an experimental experience for the exploration of the interaction mechanism among microbial communities, especially coral-associated *Vibrio* in the coral ecosystem, and potential probiotic strategy or QS regulation on further coral disease control.

## Graphical Abstract

Ecological balance among coral, microorganism and environment. Coral health was affected by the environmental changes of temperature raising, ocean acidification and coastal pollution, and microbial interaction in coral holobiont. 
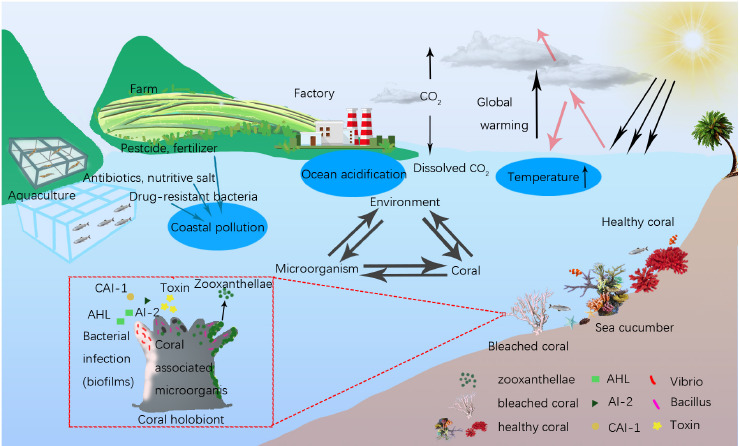

## 1. Introduction

Coral reef ecosystems possess the highest productivity and biodiversity among marine ecosystems; however, due to changes in environmental conditions, such as climate change and ocean acidification, the ecological balance among coral, microorganisms, and the environment has been affected (Stuart-Smith et al., 2018). In addition, the coastal pollution caused by human activities also led to ecological imbalance, especially the microorganism shift, resulting in the increasingly serious problems of coral degradation (Putnam and Gates, 2015) and arousing the wide attention of researchers.

The coral-associated microorganisms play important roles to maintain the health of coral holobiont (Rosenberg et al., 2009; Kang et al., 2022), which assists the coral host to adapt to the environmental changes by a shift of the symbiotic microorganism community. However, the changes in environmental conditions might lead to the disorder of the microorganism community, including the dissociation of algal symbionts from the coral symbiotic micro-ecosystem, which normally causes coral bleaching, or a shift in the bacterial community, which results in unbalanced nutrition metabolism, which causes coral disease, or the increasing richness of opportunistic pathogens, which causes bacterial infection (Mao-Jones et al., 2010). Although the proposed “bacterial bleaching” hypothesis (Kushmaro et al., 1996) is still controversial (Ainsworth et al., 2008), there have been continuous reports in recent years to prove the correlation between bacterial infection and coral disease (Ziegler et al., 2019), which highlighted the hypothesis that coral disease might be caused by the synergistic effects from one or more pathogenic bacteria. Moreover, marine animals such as fish, shrimp, sea cucumbers, and even starfish in coral reefs play the role of intermediate hosts of pathogens, increasing the probability of bacterial infection during their movement.

Coral bleaching is often accompanied by the abnormal structure of symbiotic microorganisms (Mhuantong et al., 2019; Mohamed et al., 2022). Almost all bacterial pathogens causing coral diseases are opportunistic pathogens, which produce virulence factors as a response to the changes in environmental conditions such as temperature and pH, or the interaction among bacteria during colonization competition, resulting in coral diseases (Kimes et al., 2012). Although *Vibrios* had been reported as the main kind of opportunistic pathogens to cause coral diseases, including *V. coralliilyticus, V. natriegens, V. parahaemolyticus, and V. shilonii* (Ushijima et al., 2014; Li et al., 2017; Ahmed et al., 2022), the relationships between other *Vibrio* sp. and coral health remained unknown, and their infection pathway and the pathogenic mechanism remained unknown.

In the previous study, a significantly increased abundance of *V. fortis* had been found in the microbial symbionts in *Porites lutea* with pigment abnormalities in Lembeh Strait, North Sulawesi, Indonesia, which indicated that *V. fortis* may be involved in the bacterial infection and caused the coral inflammatory reaction (Ou et al., 2018). To verify this inference, and also to study the potential interaction of microorganism community caused by the *V. fortis* colonization, in this study, we carried out the *Vibrio* infection experiments on the laboratory-based model of *Seriatopora guttatus* and *Pocillopora damicornis*, both of which are fast-growing and easy-reproducing coral species, using a *V. fortis* strain from sea cucumber from coral reef area and a marine-source quorum quenching (QQ) enzyme, YtnP, with positive AHL degradation activity (Sun et al., 2021) to inhibit the pathogenicity of *V. fortis*, to investigate the relationship between extrinsic *V. fortis* and coral health and to reveal its effects on coral-associated microorganism community by bacterial interaction.

## 2. Materials and methods

### 2.1. Bacteria, coral, and QQ enzyme reagent

The *Vibrio fortis* strain S10-1 (MCCC 1H00104) was isolated from sea cucumber from the coral reef of Lembeh Strait, North Sulawesi, Indonesia, and kindly provided by the Third Institute of Oceanography, MNR (Xiamen, China). The corals *S. guttatus* and *P. damicornis* were artificially bred and kindly provided by the coral conservation laboratory of the Third Institute of Oceanography. The YtnP enzyme was prepared by this lab in a previous study (Sun et al., 2021).

### 2.2. Bacterial culture

The S10-1 strain was inoculated in a 100-ml of Marine Broth 2216 (BD, USA) media and incubated at 30°C with shaking at 180 rpm to obtain the bacterial growth curve. The overnight culture of S10-1 at exponential growth phase, with an absorbance of about 0.50 at the optical density (OD) at 595 nm (approximate 1.0 × 10^8^ CFU/ml), was resuspended using the same volume of seawater to prepare a bacterial solution for the further experiment of bacterial infection.

### 2.3. Coral culture and bacterial infection

**FIGURE 1:**
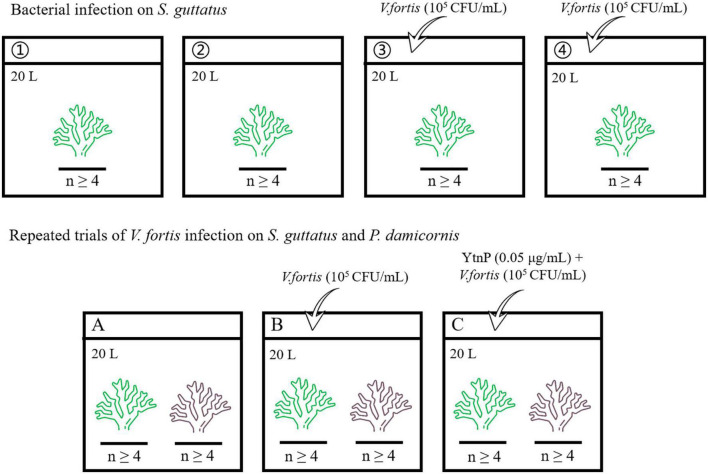
Flowchart showing the experiment overview.

The aquaria with the size of 60 × 45 × 45 cm (length × wide × high) were prepared for coral culture, with 20 L pump-recycling seawater at a salinity of 33.3‰, a protein skimmer to remove dissolved organic compounds, and a dimmable LED light to simulate the diurnal rhythm (light on between 6 a.m. and 6 p.m.). The live stone was moved from the coral conservation laboratory, randomly separated into pieces, and pre-cultured individually in the aquaria for 7 days at 25°C to maintain clean and stable water quality for coral growth. The corals were cut into branches with a length of about 80 mm, randomly separated, and placed in the aquaria for the subsequent experiment, where each aquarium contains at least four coral branches for biological repetition. The overview of the bacterial infection experiment is shown in Figure 1 and described later.

#### 2.3.1. Bacterial infection on *S. guttatus*

The coral branches of *S. guttatus* in each aquarium were immersed in 20 ml of the above prepared *Vibrio* solution for 10 min for bacterial infection (first diluted with 180 mL of seawater in a beaker to ensure that all of the coral branches could be immersed, then poured into the aquarium to make the inoculation with a final concentration of about 10^5^ CFU/mL), with the same volume of seawater as a negative control in blank groups. The treated coral branches were continuously cultured at 25°C. Each treatment was performed in duplicate.

#### 2.3.2. Repeated trials of *V. fortis* infection on *S. guttatus* and *P. damicornis* with QQ treatment

The coral branches of *S. guttatus* and *P. damicornis* were placed in the same aquarium and performed the earlier *V. fortis* inoculation with a final concentration of 10^5^ CFU/ml, using the control groups treated with *V. fortis* and an additional 2 mL of purified YtnP enzyme (working concentration at 0.05 μg/ml) to inhibit the *V. fortis* pathogenicity as a control, and the same volume of seawater as a negative control in the blank. The treated coral branches were continually cultured at 25°C.

### 2.4. Data collection and sample preparation

The condition of coral health was recorded by a digital camera and judged by the marking of the color scale (Rosado et al., 2019), according to the PANTONE Color Manager software V2.2.0. The images of corals in each group were taken using a digital camera (Canon EOS 600D with 50 mm lens, Japan), using the parameter of aperture at F/3.5, the exposure time for 1/50 s, and the sensitivity at ISO 200.

The Chlorophyll Fluorescence Quantum Yield (Qy, Fv’/Fm) of coral was determined by the mean value of the measurement in light at the tip and middle of coral branches, using pulse amplitude modulated (PAM) fluorimetry (FluorPen FP100, Czech) in triplicate. The measurement was taken at a fixed time every day before light off for the first *Vibrio* infection and performed every 4 h during the experiment in repeated trials. Statistical differences of Qy analyses were determined using one-way ANOVA.

The coral branches, about 5 g of live stone, and 500 ml of water from each group were sampled after bacterial infection. The sampled coral branches and live stones were immediately frozen by liquid nitrogen and crushed into powder using a pre-sterilized mortar and pestle, and the water was filtered by the 0.22 μm membrane (Millipore, USA) to collect the microorganisms on the filter membrane. The treated coral, stone, and water samples containing the symbiotic microorganism were then stocked at −80°C for further analysis.

### 2.5. DNA extraction and amplification

Total genomic DNA was extracted from samples using the MP FastDNA Spin kit for Soil (MP Biomedicals, USA). The DNA extract was checked on 1% agarose gel, and DNA concentration and purity were determined with NanoDrop 2000 UV-vis spectrophotometer (Thermo Scientific, USA) and used as a template DNA to amplify the hypervariable region V3–V4 of the bacterial 16S rRNA and Symbiodiniaceae ITS2 sequence with the primer pairs (listed in Table 1) by PCR using Phusion^®^ High-Fidelity PCR Master Mix kit (NEB, UK). The PCR amplification of 16S rRNA was performed as follows: initial denaturation at 95°C for 3 min, followed by 30 cycles of denaturing at 98°C for 30 s, annealing at 55°C for 30 s, and extension at 72°C for 15 s, and single extension at 72°C for 5 min, and end at 4°C. The PCR products containing bands of 400–450 bp were operated using electrophoresis on 2% agarose gel for detection, and the samples were extracted from the 2% agarose gel and purified using the DNA Gel Extraction Kit (Omega, China) according to the manufacturer’s instructions and quantified using a NanoDrop 2000 spectrophotometer (Thermo Scientific, USA).

**TABLE 1:** Primer pairs tested.

Primer	Sequence	References
338F	5′-ACTCCTACGGGAGGCAGCAG-3′	Feng et al., 2020
806R	5′-GGACTACHVGGGTWTCTAAT-3′	Feng et al., 2020
ITS-DINO	5′-GTGAATTGCAGAACTCCGTG-3′	Hume et al., 2018
ITS2Rev2	5′-CCTCCGCTTACTTATATGCTT-3′	Hume et al., 2018

### 2.6. High-throughput sequencing and microbial diversity analysis

The purified amplicons were pooled in equimolar and paired-end sequenced on an Illumina MiSeq PE300 platform/NovaSeqPE250 platform (Illumina, San Diego, USA) according to the standard protocols by Majorbio Bio-Pharm Technology Co., Ltd. (Shanghai, China).

The raw bacterial 16S rRNA and Symbiodiniaceae ITS2 sequencing reads were demultiplexed, quality filtered by fastp version 0.20.0 (Chen et al., 2018), and merged by FLASH version 1.2.7 (Magoè and Salzberg, 2011) with the following criteria: (i) the 300 bp reads were truncated at any site receiving an average quality score of <20 over a 50 bp sliding window, and the truncated reads shorter than 50 bp and reads containing ambiguous characters were discarded; (ii) only overlapping sequences longer than 10 bp were assembled according to their overlapped sequence. The maximum mismatch ratio of the overlap region is 0.2. The reads that could not be assembled were discarded; (iii) samples were distinguished according to the barcode and primers, and the sequence direction was adjusted with exact barcode matching or a maximum of two nucleotide mismatch in primer matching.

Operational taxonomic units (OTUs) with a 97% similarity cutoff were clustered using the UPARSE version 7.1 (Edgar, 2013), and the chimeric sequences were identified and removed. The taxonomy of each OTU representative sequence was analyzed by RDP Classifier version 2.2 (Wang et al., 2007) according to the SILVA ribosomal RNA database and algal symbionts Genomic resource database (Shi et al., 2021), using a confidence threshold of 0.7. Alpha diversity index, including observed OTUs, and richness estimators, such as Ace, Chao, Shannon, and Simpson indices, were calculated based on the frequency of OTUs and genera in the assigned sequence collections after rare sequences were removed. The OTUs were analyzed by Student’s *t*-test to compare the difference of the abundance on the genus level.

## 3. Results

### 3.1. Effect of *V. fortis* on the coral health

**FIGURE 2:**
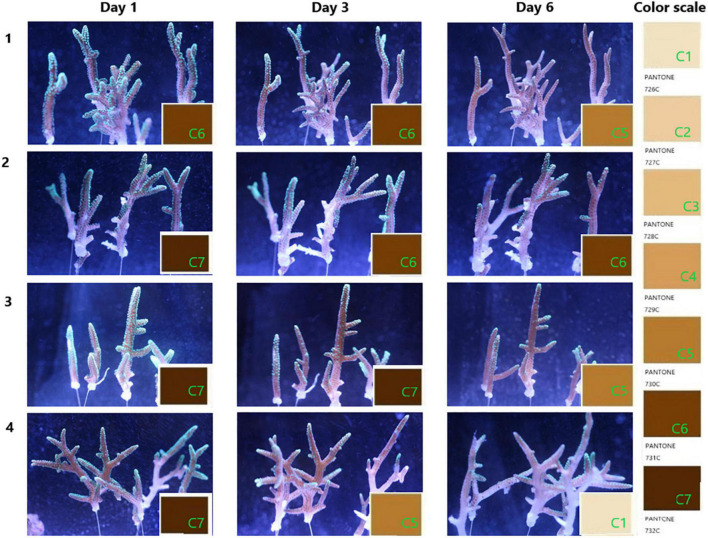
Progress of coral bleaching. The coral branches were cultured in pump recycling water with a salinity of 33.3‰ at 25°C under the simulated diurnal rhythm by LED light. Groups 1 and 2 are the corals treated with seawater in the blank; groups 3 and 4 are the corals infected by the overnight culture of *Vibrio fortis* S10-1. The images present the progress of coral bleaching judged by the color scale marked from C1 to C7, referenced from the PANTONE color system of 726–732C.

The condition of coral health is shown in Figure 2. After the inoculation of S10-1 for about 30 h, the corals in blank groups as the control were still observed in good condition, while the infected ones repeated in Groups 3 and 4 were observed with less tentacles extending for predation (Day 3). The second inoculation of *Vibrio* on Day 5 caused the symptoms to be more obvious on the second day (Day 6), and the images showed that Group 3 infected with *Vibrio* was observed with bleaching from C7 to C5, and Group 4 observed more serious bleaching from C5 to C1, accompanied with peeling of coral tissue. The damages mainly occurred starting from the middle of coral branches to the tip ends, while the coral polyps still seemed to be in good condition at the tip ends.

### 3.2. Photosynthetic function of coral holobiont under *V. fortis* infection

**FIGURE 3:**
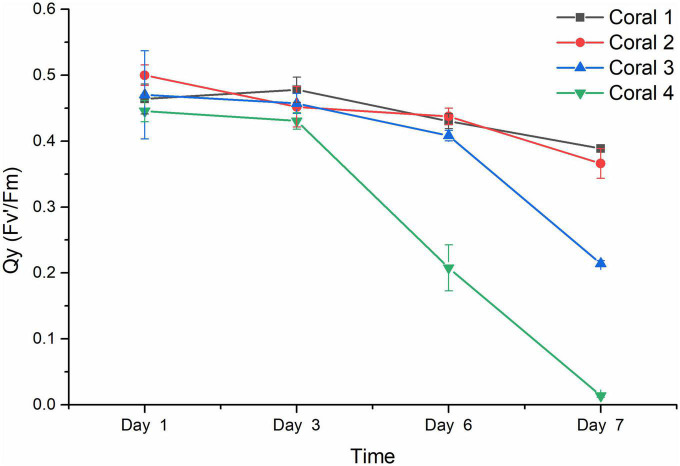
Chlorophyll fluorescence quantum yield (Fv’/Fm) of coral holobiont. The Qy (Fv’/Fm) was measured using PAM fluorimetry at 3 points of coral middle parts and 3 points of coral tip ends, to obtain the average value and standard deviation of each coral branch. Corals 1 and 2 represented the corals treated with seawater in the blank; corals 3 and 4 represented the corals infected by *V. fortis* S10-1. The Qy of coral 1 is present by █ in black line, coral 2 by • in red line, coral 3 by ▲ in blue line, and coral 4 by ▼ in green line.

The results from the visual response of the coral holobiont health in each treatment were confirmed by the chlorophyll fluorescence quantum yield (Fv’/Fm) measured by PAM fluorometry to indicate the algal symbionts’ photosynthetic function. As shown in Figure 3, the Qy values from duplicated blank groups were similar, ranging between 0.50 and 0.40 in the first 6 days and remaining above 0.35 on Day 7 (*P*-value was 0.96857), while the Qy values from duplicated *Vibrio* infection groups also did not present a significant difference (*P*-value was 0.37669), with coral 3 being observed with a decreasing ratio of 13.17% after 6 days and further decreasing to about 0.21 on Day 7, while coral 4 present more sensitive to the *Vibrio* infection, decreased rapidly from healthy status at about 0.45 to an obvious bleaching status at Qy valve of 0.208 on Day 6 than 0.014 on Day 7. According to the results of statistical analysis, the *Vibrio* infection significantly affected the photosynthetic function of coral holobiont with a *P*-value of 0.00491.

### 3.3. Verification of *V. fortis* as a potential pathogen to coral disease

**FIGURE 4:**
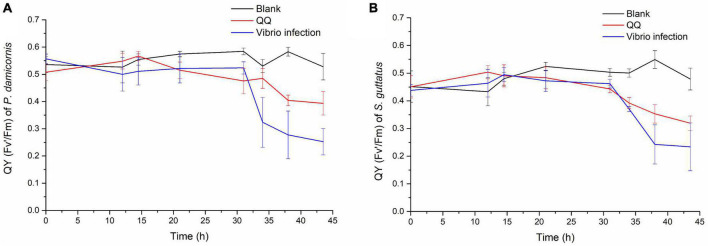
Photosynthetic function of coral holobiont under bacterial infection and QQ treatment. The Qy (Fv’/Fm) of *P. damicornis* **(A)** and *S. guttatus* **(B)** was measured using PAM fluorimetry at 3 points of coral middle parts and 3 points of coral tip ends, to obtain the average value and standard deviation of each coral branch. The Qy from the *V. fortis* infected groups are presented in blue line, those infected groups with additional treatment of QQ enzyme are presented in red line, and the blank groups in black line.

Later on, the S10-1 strain was isolated from the infected *S. guttatus* branches. According to Koch’s rule, to verify the actual causative agent of *V. fortis*, subsequent infection experiments were carried out and inoculated the isolated S10-1 on both *S. guttatus* and *P. damicornis*; in addition, the QQ enzyme was used to inhibit the pathogenicity of S10-1 as control. As shown in Figure 4, the two species of coral were observed with a significant decrease in Qy value accompanied by obvious bleaching, after the inoculation of re-isolated S10-1 for 30 h. Interestingly, the corals in QQ-treated groups were observed with a reduction tendency but far better than that of infected groups, while the corals in blank groups remained in good condition. The results demonstrated that the infection of *V. fortis* again caused coral bleaching after bacterial inoculation, confirming the hypothesis that *V. fortis* was highly responsive to coral bleaching and was a potential pathogen related to coral health. In addition, the positive effects of QQ also indicated the probability of quorum sensing (QS) inhibition as a potential strategy for coral disease control.

### 3.4. Diversity and richness of total symbiotic bacteria

The diversity and richness of symbiotic bacteria from *S. guttatus* were further analyzed by the Alpha index, as shown in Table 2. The coverage of OTU from each sample was in the range of 98.79 to 99.95%, which indicated good recovery and reliable reflection of the symbiotic bacterial community of the tested samples.

**TABLE 2:** Alpha diversity index analysis of symbiotic bacteria in the coral culture system.

Sample	OTU number	Ace	Chao	Shannon	Simpson	Coverage
coral_1	30855	249.1679	256.5556	1.3627	0.6033	99.91%
coral_2	31511	127.4174	157.7500	0.8403	0.7541	99.94%
coral_3	31731	407.2778	409.1579	2.9992	0.2437	99.95%
coral_4	32228	411.5785	414.5000	3.3771	0.1833	99.95%
water_1	42886	533.0147	499.2113	1.5774	0.4193	99.68%
water_2	37960	467.1878	463.1000	2.6786	0.1588	99.70%
water_3	36706	531.7272	488.5789	2.2264	0.2431	99.68%
water_4	33842	246.4882	238.0000	1.4988	0.3575	99.84%
stone_1	37081	1856.7257	1824.1246	5.7499	0.0103	99.24%
stone_2	30049	1738.6738	1704.2672	5.4152	0.0190	98.84%
stone_3	31703	1867.3839	1844.3333	5.5812	0.0118	98.79%
stone_4	38012	1601.2413	1580.1416	5.5155	0.0104	99.27%

The community of coral symbiotic bacteria was observed with a remarkable difference between the healthy corals and the *Vibrio*-infected ones; the infection of *V. fortis* increased the richness as indicated by the higher indices of Ace and Chao and enriched the diversity as indicated by the higher Shannon index and lower Simpson index. In contrast, the planktonic bacteria in the water sample of healthy corals present higher richness than that of diseased corals and showed similar diversity between groups. In addition, the richness and diversity of the bacterial community in live stones were rarely affected by the additional *V. fortis* and showed no significant difference.

### 3.5. Structure of microbial community in the coral culture system

**FIGURE 5:**
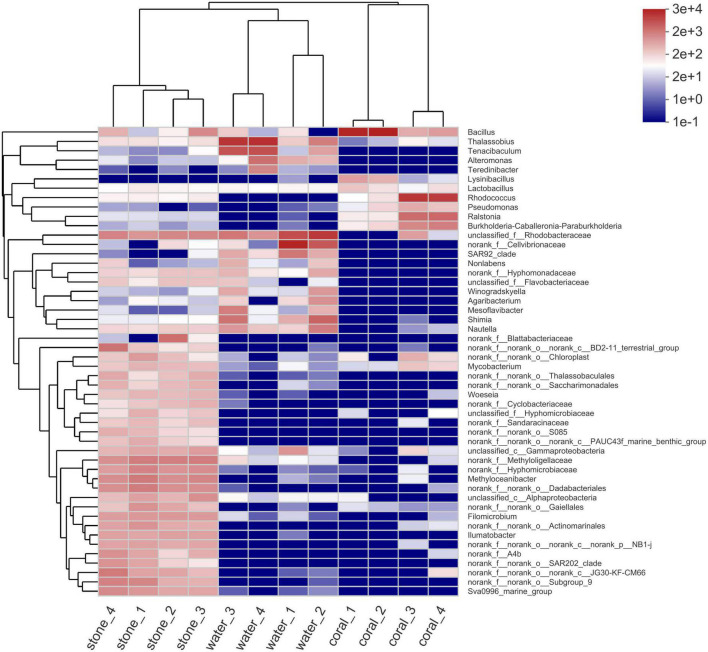
Community species abundance clustering heatmap of coral culture system on the genus level. The horizontal clustering is sample information, and the vertical clustering is species information. The left clustering tree is the species clustering tree and the upper clustering tree is the sample clustering tree. The middle is the abundance heatmap.

The microbial diversity in *S. guttatus* of each treatment group was analyzed. As shown in the community species abundance clustering in Figure 5, the upper clustering tree analyzed using the Bray–Curtis algorithm demonstrated a similarity and consistent tendency on the species abundance of the bacterial community in the duplicates, which indicated good reliability of the experiments. The results also indicated the relevance of bacterial community in the coral culture system, indicating that the *Vibrio* infection changed the abundance of the bacterial community in the symbiotic status of coral holobiont and planktonic status in seawater and that there was no significant difference among stones exposed under immersion of *Vibrio*, indicating the ability of self-regulation and stronger colonization resistance, which was probably acted by the abundant microorganisms adhered on the stones.

#### 3.5.1. Community of coral symbiotic microorganisms

**FIGURE 6:**
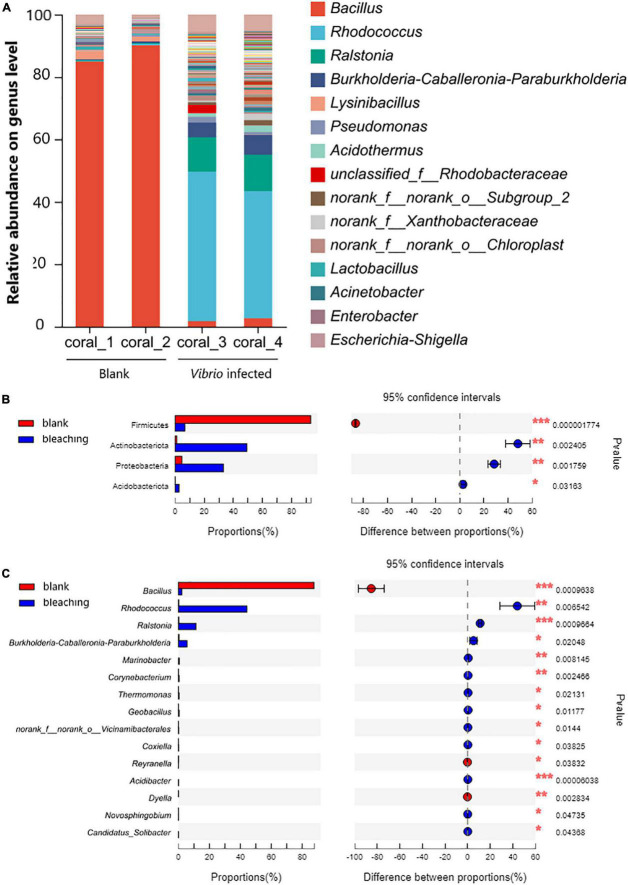
Community composition of coral symbiotic bacteria on the genus level. The total genomic DNA of coral symbiotic microorganisms from each group performed high-throughput sequencing on the 16S rRNA. The taxonomy of each OTU representative sequence was analyzed by RDP classifier version 2.2 and classified on the genus level. The OTUs were analyzed by student’s *t*-test to compare the difference in abundance between groups: **(A)** is the microbial structure presented by relative community abundance; **(B)** is the phyla with a significant difference in abundance; and **(C)** is the top 15 genera with a significant difference in abundance were listed from higher abundance to low. Results of statistical analysis are presented by the significant difference indicated by * where 0.01 ≤ *P* ≤ 0.05, ** where 0.001 ≤ *P* ≤ 0.01, and *** where *P* ≤ 0.001.

The structure of the microbial community in corals is shown in Figure 6. The results of OTU analysis showed that the microbial community composition was significantly different between the groups with different treatments. On the phylum level, the microbial community of corals suffered from exogenous *V. fortis* infection which led to a significant decrease in Firmicutes from 93.14 to 6.624% (*P*-value lower than 0.001) but an increase in the abundance of Actinobacteriota from 1.085 to 49.26% (*P*-value was 0.002405), Proteobacteria from 4.68 to 33.19% (*P*-value was 0.001759), and Acidobacteriota from 0.08413 to 2.653% (*P*-value was 0.03163).

On the genus level, after infection with *V. fortis*, the abundance of *Bacillus* in the symbiotic microorganisms of bleaching coral decreased significantly from 87.57% in the blank group to only 2.273% in bleaching corals (*P*-value was 0.0009638), mostly contributed by more abundant *B. circulans* and *B. aryabhattai* and less abundant *B. subtilis*, *B. anthracis*, and *B. infernus*, and the abundance of *Rhodococcus* with a *P*-value of 0.006542 (44.26% in bleaching coral and 0.2874% in the blank group, mostly identified as *R. erythropolis*), *Ralstonia* with a *P*-value of 0.0009664 (11.35% in bleaching corals and 0.3126% in the blank group, affected by *R. pickettii* and *R. insidiosa*), and *Burkholderia–Caballeronia–Paraburkholderia* with a *P*-value of 0.02048 (5.606% in bleaching corals and 0.4076% in the blank group, mostly constituted by *Paraburkholderia fungorum*) increased significantly.

#### 3.5.2. Community of planktonic bacteria in water

**FIGURE 7:**
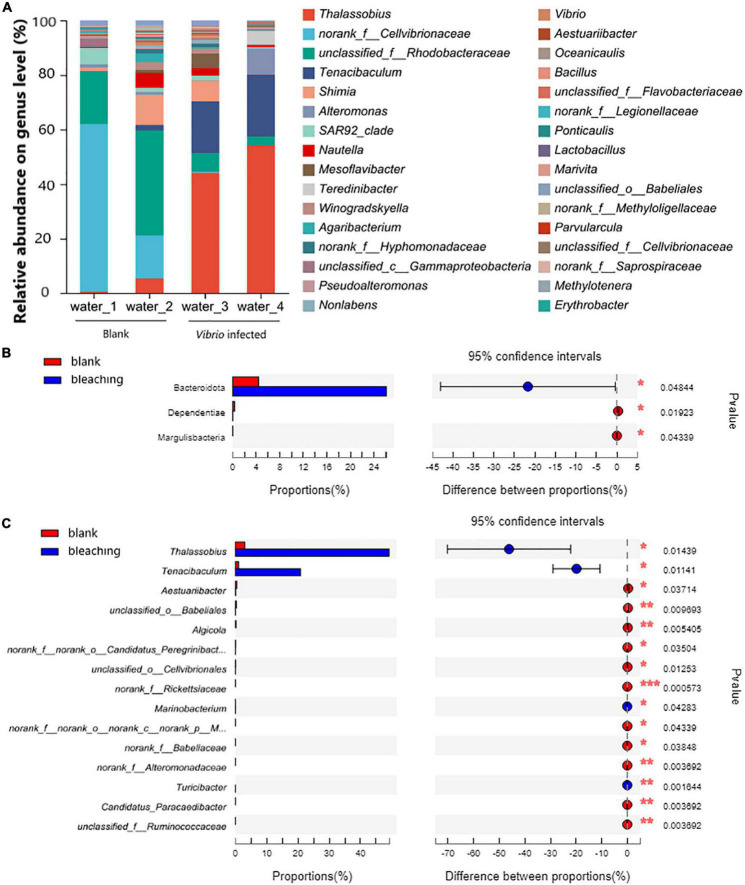
Community composition of planktonic bacteria in water on genus level. The total genomic DNA of planktonic bacteria in water from each group performed high-throughput sequencing on the 16S rRNA. The taxonomy of each OTU representative sequence was analyzed by RDP Classifier version 2.2 and classified on the genus level. The OTUs were analyzed by student’s *t*-test to compare the difference in abundance between groups: **(A)** is the microbial structure presented by relative community abundance; **(B)** is the phyla with a significant difference in abundance; and **(C)** is the top 15 genera with a significant difference in abundance which were listed from higher abundance to low. Results of statistical analysis are presented by the significant difference indicated by * where 0.01 ≤ *P* ≤ 0.05, ** where 0.001 ≤ *P* ≤ 0.01, and *** where *P* ≤ 0.001.

The planktonic bacteria in the culture water were also analyzed as shown in Figure 7. On the phylum level, Proteobacteria took the highest proportion in the community and showed no significant difference after infection (94.21% in blank and 73.1% after infection, the *P*-value was 0.05276); the *Vibrio* infection caused significantly increasing abundant Bacteroidota from 4.402 to 26.09% (*P*-value was 0.04844); decreasing abundant Dependentiae from 0.3424 to 0.01805% (*P*-value was 0.01923) and Margulisbacteria with 0.02686% in the blank group and non-detected in *Vibrio*-infected groups (*P*-value was 0.04339).

On the genus level, the results of OTU analysis demonstrated a significantly increased abundance of *Thalassobius* and *Tenacibaculum*, and took as much as 49.15 and 20.83% in the *Vibrio*-infected culture water, while there were only 2.995 and 1.039% in the blank group (*P*-values were 0.01439 and 0.01141, respectively). It was also observed that with abundant unclassified genera from the Rhodobacteraceae family (5.083% in the *Vibrio*-infected group and 28.91% in the blank group) and *Shimia* (3.862% in the *Vibrio*-infected group and 6.152% in the blank group) present in the culture water, there was no significant difference between groups (*P*-values were 0.1342 and 0.7453, respectively).

#### 3.5.3. Proportion of *Vibrios* in the coral culture system

**FIGURE 8:**
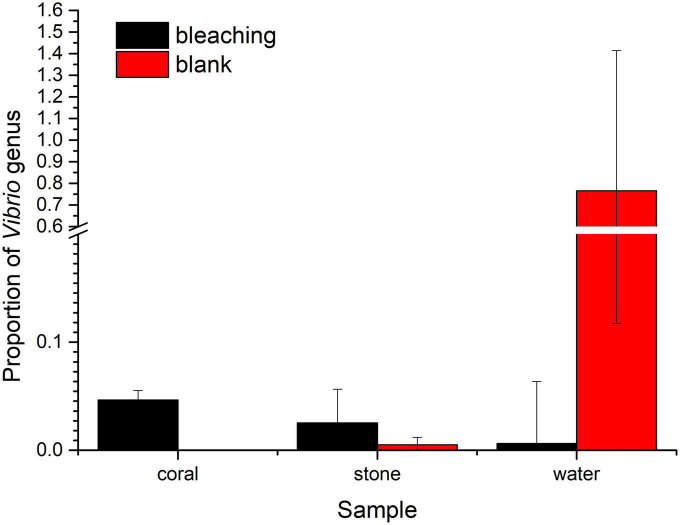
Proportion of *Vibrios* in each fraction of coral culture system. The taxonomy of each OTU representative sequence from the high-throughput sequencing result was analyzed by RDP classifier version 2.2 and classified on genus level. The proportion of the OTUs representing the *Vibrio* genus is presented in bar charts, where the samples in the blank group are presented in red, and those in S10-1-infected groups are presented in black. The error bars indicate the value of standard deviations.

As for the *Vibrio* sp., the proportion of *Vibrios* in each fraction is shown in Figure 8. The abundance of *Vibrio* was 0.006206% in bleaching corals, while that was non-detected in the blank group, including OTU3764 whose sequence was 100% matched to the 16S rRNA sequence of S10-1 (which is consistent with the result of isolation of S10-1 from infected *S. guttatus*) and other four unidentified *Vibrio* sp. represented by OTU3690, OTU3768, OTU3779, and OTU38. A higher proportion of *Vibrios* was also observed in the stones under *Vibrio* infection; however, the *Vibrio* abundance of water in the S10-1 infected group was significantly lower than that of the blank group, indicating that the change in the bacterial community and coral health was not directly caused by the *Vibrios* under planktonic status, and probably affected by the symbiotic status in corals.

### 3.6. Effect of *Vibrio* infection on the algal symbionts

**FIGURE 9:**
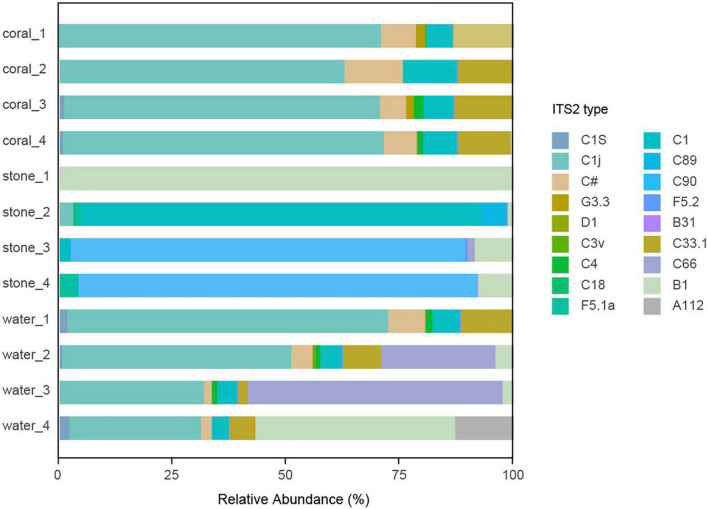
Community component of algal symbionts in the coral culture system. The total genomic DNA from each group performed high-throughput sequencing using the primer pair of ITS-DINO and ITS2Rev2. The taxonomy of each OTU representative sequence was analyzed according to the algal symbionts’ genomic resource database and classified by ITS2 type.

The community of algal symbionts was also analyzed to assess the effects of *Vibrio* infection as shown in Figure 9. The community of algal symbionts was observed with the most components of *Cladocopium* sp. in coral holobionts and very few *Breviolum* sp. and *Fugacium* sp. in live stone and seawater. No significant difference in community abundance and diversity was observed among the groups in coral holobiont, which indicated the rare effect of *Vibrio* on the algal symbionts, and therefore verified that the phenomenon of bleaching might be caused by the bacterial infection reasoned by *Vibrio* and secondary disorder of bacterial community.

## 4. Discussion

### 4.1. Potential pathogenicity of *Vibrio* to coral health

The role of *Vibrios* in coral diseases was still limitedly known (Munn, 2015), though there are some *Vibrio* species had been identified as opportunistic pathogens to cause coral disease, there is disagreement about whether they should be regarded as primary causing agents, as some *Vibrios* appeared to contribute to nitrification (Thurber et al., 2009) or involved in defense against pathogens (Ritchie, 2006).

Unlike the better-studied species, such as *V. parahaemolyticus* and *V. coralliformis*, *V. fortis* is still rarely reported. To the best of our knowledge, it was reported for its pathogenicity to marine animals such as shrimps (Thompson et al., 2003), sea horses (Wang et al., 2016), and sea urchins (Ding et al., 2014), as well as one of the lists of abnormal abundant coral symbiotic microbes (Ou et al., 2018). Predictably, the activity of sea cucumber carrying *V. fortis* in coral reefs area would increase the risk of bacterial infection to the coral holobiont. However, the disease development and pathogenicity mechanism of coral bleaching caused by *V. fortis* are not yet clear.

It is currently known that the pathogenicity of *Vibrio* sp. is parallelly regulated by at least three QS pathways, LuxM/LuxN-related AHLs, CqsA/CqsS-related CAI-1, and LuxS/LuxP-related AI-2; all lead to the regulation of core regulatory protein LuxO (Herzog et al., 2019) and then to the synergistic action of key regulatory proteins AphA (Lu et al., 2018) and OpaR (Zhang et al., 2016), which regulate downstream exopolysaccharide synthesis genes to affect biofilm formation and virulence relative genes to release virulence factors including hemolysin. The findings of QS signals produced by bacteria in the coral mucus layer (Li et al., 2017), the signaling molecules and active hydrolytic enzymes released by *V. shilonii* AK1 in outer membrane vesicles (Li et al., 2016), and the interference of QS regulation against the AHL-mediated opportunistic bacteria of resident microbes in bleaching’s initiation and progression (Zhou et al., 2020), in recent years, all supported the hypothesis of bacterial infection under QS regulation, which is consistent with the positive inhibition of *V. fortis* pathogenicity under QQ enzyme treatment in this study (Figure 4), and this phenomenon indicates the potential strategy of QQ on coral disease control.

### 4.2. Microbial shift and bacterial interaction behind *Vibrio* infection

In this article, the infection of *V. fortis* was designed as the only variable factor in coral health. The symptom development of coral bleaching and tissue lysis verified and strongly supported the hypothesis of “bacterial bleaching.” The findings in this study demonstrated that its infection also caused significant changes in the coral-associated microsystem, especially the increased abundance of opportunistic pathogenic bacteria, which might synergistically affect coral health.

Among the microbial community, the decreasing abundance of *Bacillus* belonging to phylum Firmicutes seems to be the most serious side effect caused by *Vibrio* infection (Figure 5). *Bacillus* had been widely accepted as the main antagonistic bacteria and used for biological control in agriculture (Jiang et al., 2018) and aquaculture (Wang et al., 2019), and recently reported as marine probiotics to increase coral resistance to bleaching (Rosado et al., 2019). *Bacillus* can produce secondary metabolites, including lipopeptides and polyketones with antibacterial, antiviral, or antitumor activities (Wu et al., 2019), and the quorum quenching (QQ) enzymes, such as AiiA (Dong et al., 2000) and YtnP (Sun et al., 2021), to degrade AHLs, inhibiting the biofilm formation and toxin release of AHLs/Lux-mediated pathogens including *V. fortis* that was known to contain an AHLs intermediated LuxM/LuxN QS pathway (Ding et al., 2014). The subsequent abnormal abundant *Bacillus*, *B. circulans* for instance, resulted from the simulated outbreaking of *V. fortis* when the inoculation of 10^5^ CFU/ml was far beyond the threshold of QS; therefore, it might further weaken the resistance of coral holobiont to the stress from pathogens (Satpute et al., 2010; Niu et al., 2014).

The increased abundance of *Rhodococcus* in phylum Actinobacteria might also be related to the decreasing abundance of *Bacillus*, because *Rhodococcus sp.* was reported with antibacterial activity against *Bacillus substilis* (Mahmoud and Kalendar, 2016). Among these, the most composition species, *R. erythropolis*, produces different types of QQ enzymes (Ryu et al., 2020) to recognize and degrade AHLs and could be used for biofouling control (Ergön-Can et al., 2017). The increasing abundance might be speculated to result from the self-regulation of the coral symbiotic microbiota after the sense of abnormally increased concentration of AHLs generated by the inoculated *V. fortis*.

The increasing abundance of the genera *Ralstonia* and *Burkholderia–Caballeronia–Paraburkholderia* in phylum Proteobacteria was probably eligible for the causal relationship of the declined abundance of *Bacillus* because of the less inhibition from the *Bacillus*-produced surfactin (Grady et al., 2019). However, the correlation between *Ralstonia* and *Burkholderia–Caballeronia–Paraburkholderia* of coral health is still unclear (Bernasconi et al., 2019). Reports are claiming that *Ralstonia* sp. is a known gram-negative phytopathogenic bacteria (Hayashi et al., 2019) and dental opportunistic pathogen (Tuttlebee et al., 2002), while the *Burkholderia–Caballeronia–Paraburkholderia*, which is mostly constituted by *Paraburkholderia fungorum*, is reported as a plant probiotic bacteria (Rahman et al., 2018) but still risky to human health (Tan et al., 2020).

In addition, considering that the planktonic bacteria in the water could flow into the gastrovascular cavity of coral along with the filter feeding, the significantly increased abundance of *Thalassobius* (also detected with higher abundance in bleaching coral but with no significant difference) and *Tenacibaculum* in water might also affect the coral health (Figure 7). *Thalassobius* belonging to the family Rhodobacteraceae has been reported as a microbial bioindicator enriched in the Stony Coral Tissue Loss Disease accompanied by *Vibrio* (Becker et al., 2021), and has been associated with invertebrate diseases (Roder et al., 2014), while the increased abundance of *Tenacibaculum* was also consistent with the findings from microbial community shift in the White syndrome-affected *Echinopora lamellosa* in aquaria (Smith et al., 2015).

Another unclassified genus belonging to the family Rhodobacteraceae and the genus *Shimia* was also detected in the planktonic bacterial community in water, though there was no significant difference observed yet. The indicator species in the coral hosts, family Rhodobacteraceae and genus *Shimia*, were observed to increase their relative abundance when corals are under stress (Casey et al., 2015; Pootakham et al., 2019) or with the emergence of *Porites* white patch syndrome accompanied with *Vibrio* (Séré et al., 2013). This phenomenon was verified in the later coral bleaching in the blank group caused by the deterioration of water quality 2 days after the experiments stopped (data not shown).

The virulence from the shift bacterial community of abundant opportunistic pathogens might not be the only reason for coral bleaching. The nutrients sources and waste products for coral holobiont are also a concern, which mainly come from the metabolism of symbiotic microbes such as carbon and nitrogen fixation (Gibbin et al., 2019) and the metabolic integration from algal symbionts (Sun et al., 2020), in such closed aquaria without extra exogenous nutrient sources. The shift in the bacterial community no doubt leads to an imbalance in the nutrient food chain in the coral holobiont (Raina et al., 2009); however, in this study, there is no direct relevance between the infection of *V. fortis* and the status of algal symbionts. The dominant population in the tested *S. guttatus* was *Cladocopium* sp., though with few relative abundances of other Symbiodiniaceae according to the reported ITS2 type (Yu et al., 2020), and no observation of *Durusdinium* sp., which was reported with stronger stress resistance (Sun et al., 2020); therefore, the findings indicated that the bleaching in this study was caused by *Vibrio* infection and the following shift of bacterial community, but not by the dissociation of algal symbionts.

To sum up, this study expands the cognition of the correlation between coral symbiotic *Vibrio* and coral health. According to the experience of other reported *Vibrio* pathogens, the outbreak and pathogenicity enhancement of *Vibrio* may be related to environmental changes (Kimes et al., 2012). The symbiotic changes caused by the outbreak of *V. fortis*, especially the increased abundance of other pathogenic bacteria, may also cause more serious damage to the coral holobiont. In addition, the reduced abundance of potential probiotics *Bacillus* under the competition of microbiota might also provide an experimental experience for probiotic strategy (Zhao et al., 2019) or QS regulation (Zhou et al., 2020) on pathogen control or resistance enhancement (Rosado et al., 2019).

## 5. Conclusion

To identify the relationship between *V. fortis* and coral health and to understand its role in bacterial infection, *V. fortis* S10-1 was designed as the only variable factor to infect the coral *Seriatopora guttatus* and *Pocillopora damicornis*. The results of color scale analysis of S10-1-infected coral branches in aquaria indicated that *V. fortis* was responsible for coral bleaching, which leads to the high probability of pathogenicity of *V. fortis* in coral. The significant reduction of photosynthetic function in coral holobiont and shift of coral symbiotic microbial community upon the infection of S10-1 provided strong evidence for the “bacterial bleaching” hypothesis. The positive effect of quorum quenching indicated the potential strategy of bacterial disease control. The infection of S10-1 led to the imbalance in the coral-associated bacterial community but had no significant effect on the algal symbionts, and this was accompanied by a significant decrease in the abundance of probiotic *Bacillus* and an increase in the abundance of *Rhodococcus erythropolis* and other opportunistic pathogens including *Ralstonia* and *Burkholderia–Caballeronia–Paraburkholderia* in the coral-associated community, as well as increased abundance of *Shimia* and other unclassified genus in family Rhodobacteraceae in the planktonic bacterial community in water. The study provided experimental experience on corals in aquaria for the exploration of the interaction among coral-associated microbial communities in coral relative micro-ecosystem and revealed the potential probiotic strategy or QS regulation on pathogen control for coral health.

## Data availability statement

The datasets presented in this study can be found in online repositories. The names of the repository/repositories and accession number(s) can be found below: https://doi.org/10.6084/m9.figshare.21707861.v1.

## Author contributions

XS contributed to the conception of the study and drafted the manuscript. YL and XZ contributed to the design of the experiment of bacterial challenge and culture of corals. QY, NX, ZT, SW, and YJ performed the experiments. HZ helped the analysis of the ITS2 type of Symbiodiniaceae. XZ, HM, and DO helped the analysis with constructive discussions. XS and DO offered the funding acquisition. All authors reviewed the results and approved the final version of the manuscript.
